# Erythropoietin promotes M2 macrophage phagocytosis of Schwann cells in peripheral nerve injury

**DOI:** 10.1038/s41419-022-04671-6

**Published:** 2022-03-16

**Authors:** Prem Kumar Govindappa, John C. Elfar

**Affiliations:** Department of Orthopaedics and Rehabilitation, Center for Orthopaedic Research and Translational Science (CORTS), The Pennsylvania State University College of Medicine, Hershey, PA 17033 USA

**Keywords:** Neurodegeneration, Somatic system

## Abstract

Following acute sciatic nerve crush injury (SNCI), inflammation and the improper phagocytic clearance of dying Schwann cells (SCs) has effects on remodeling that lead to morbidity and incomplete functional recovery. Therapeutic strategies like the use of erythropoietin (EPO) for peripheral nerve trauma may serve to bring immune cell phagocytotic clearance under control to support debris clearance. We evaluated EPO’s effect on SNCI and found EPO treatment increased myelination and sciatic functional index (SFI) and bolstered anti-apoptosis and phagocytosis of myelin debris via CD206^+^ macrophages when compared to saline treatment. EPO enhanced M2 phenotype activity, both in bone marrow-derived macrophages (BMMØs) and peritoneal-derived macrophages (PMØs) in vitro, as well as in PMØs in vivo. EPO increased efferocytosis of apoptotic sciatic nerve derived Schwann cells (SNSCs) in both settings as demonstrated using immunofluorescence (IF) and flow cytometry. EPO treatment significantly attenuated pro-inflammatory genes (IL1β, iNOS, and CD68) and augmented anti-inflammatory genes (IL10 and CD163) and the cell-surface marker CD206. EPO also increased anti-apoptotic (Annexin V/7AAD) effects after lipopolysaccharide (LPS) induction in macrophages. Our data demonstrate EPO promotes the M2 phenotype macrophages to ameliorate apoptosis and efferocytosis of dying SCs and myelin debris and improves SN functional recovery following SNCI.

## Introduction

Traumatic peripheral nerve injury (TPNI) causes long-term morbidity owing to inflammation at the injury site [1, 2], which is characterized by a tightly controlled initial pro-inflammatory M1 macrophage phenotypic response followed by an anti-inflammatory M2 resolution phase [3, 4]. Some previous work suggests erythropoietin (EPO) modulates this type of inflammation and shifts macrophages from an M1 to M2 phenotype that facilitates phagocytosis [5, 6]. Macrophages deficient in EPO signaling have impaired phagocytosis, which results in chronic inflammation in vivo in mouse models of diet-induced obesity and lupus-like autoimmune disease [6, 7].

Nerve crush injury destroys axons and Schwann cells (SCs) that must be cleared to allow surviving SCs to de-differentiate and divide and guide the regeneration process [8, 9]. In TPNI, macrophage clearance of dying SCs and myelin debris serves to control secondary necrosis in advance of nerve regeneration [3, 4]. We hypothesized that a role in supporting M2 phenotype in macrophages after sciatic nerve crush injury (SNCI) may explain our previously published findings that EPO improves function after SNCI. This would be true if EPO directly altered macrophage phagocytosis of apoptotic SCs and would manifest in the capacity for phagocytic clearance in conjunction with the expression of M2 phenotype markers in macrophages following SNCI.

In the present study, we show that EPO treatment after SNCI significantly attenuated apoptosis which supports M2 macrophage phagocytosis of myelin debris in the nerve and leads to myelo- and neuro-protection. EPO also activated M2 phenotype engulfment at the injury site and promoted M2 phenotype in vitro by attenuating expression of pro-inflammatory genes (IL1β, iNOS, and CD68) and augmenting anti-inflammatory genes (IL10 and CD163) as well as the cell-surface marker (CD206). This was also demonstrable by stressing macrophages with lipopolysaccharide (LPS), where EPO treatment significantly enhanced M2 phenotype macrophage efferocytosis of apoptotic sciatic nerve derived Schwann cells (SNSCs) in vitro and in vivo in mice pretreated with EPO.

## Materials and methods

### Animals

All animal experiments that conform to the protocols were approved by the Institutional Animal Care and Use Committee (IACUC) at The Pennsylvania State University College of Medicine, Hershey, PA. Ten-week-old male C57BL/6 J mice weighing 25 ± 3 g were obtained from Jackson Laboratories (Bar Harbor, ME).

### Mouse model of sciatic nerve crush injury

SNCI was performed as described in our previous studies [10, 11]. Briefly, after intraperitoneal (IP) ketamine (100 mg/kg)/xylazine (10 mg/kg) anesthesia, under a stereo microscope, the SN was exposed, and crush injury was performed ~3 mm proximal to the SN trifurcation using calibrated forceps (5 mm tip width; 18-1107, Miltex Instruments) for 30 s [12]. The skin was closed with surgical staples and post-operative slow-release buprenorphine (0.05 mg/kg) was given subcutaneously to all animals as an analgesic. The experimental animals (*n* = 7 animals/group) were randomly assigned to Sham (normal saline, 0.1 ml/mouse), SNCI (normal saline, 0.1 ml/mouse), and SNCI with EPO (5000 IU/kg; Epoetin alfa, PROCRIT^®^) groups. EPO was given intraperitoneally immediately after surgery and on post-surgery day 1 and day 2. Post-injury functional recovery was assessed by walking track analysis (WTA) on days 3 and 7. Mice were euthanized on either post-injury day 3 or 7, and SN was harvested from the ipsilateral hindlimbs for apoptosis and efferocytosis analysis using immunofluorescence (IF) staining.

### Sciatic functional index

To evaluate in vivo sciatic function index (SFI), WTA was performed on post-SNCI days 3 and 7 as described in our previous work [10, 11]. SFI was calculated using three parameters of footprints: (1) toe spread (TS, first to the fifth toe), (2) total print length (PL), and (3) intermediate toe spread (IT, second to the fourth toe) and the following formula: SFI = −38.3{(EPL-NPL)/NPL} + 109.5{(ETS-NTS)/NTS} + 13.3{(EIT-NIT)/NIT}−8.8, where E for experimental (injured) and N for normal (contralateral uninjured) sides.

### Sciatic nerve processing and in situ cell death detection study

SN processing was performed as described previously [10]. Briefly, SN was fixed in 4% paraformaldehyde (#J19943-K2; Thermo Scientific) solution overnight and washed with 70% alcohol three times for 30 min, and then embedded in paraffin. The serial 5-μm-thick longitudinal sections of SN were taken from the embedded blocks using a microtome (#RM2235; Leica). Cell death detection was performed using terminal deoxynucleotidyl transferase dUTP nick end labeling (TUNEL) and propidium iodide (PI) co-staining as previously described with slight modifications [13]: (1) paraffin-embedded SN tissue sections were heated on a slide warmer for 45 min at 60 °C, (2) tissue sections were deparaffinized (xylene) and serially rehydrated (alcohol), (3) permeabilization of tissues was performed using 1% Triton X-100 and 5 mM sodium citrate buffer (pH6.0) in distilled water for 10 min at 4 °C, (4) TUNEL reaction mixtures were instantly prepared as per company specifications (#11684795910, Roche) and incubated with tissue sections for 60 min in a humidified chamber at 37 °C. Tissue sections were washed with 1xDPBS three times at 5 min. PI staining (#4830-01-K; TREVIGEN) was performed as per company specifications for 10 min at room temperature. Finally, tissue sections were washed with 1XDPBS, and coverslips were mounted with ProLong^TM^ Gold anti-fade reagent with DAPI (#P36935; Invitrogen) and examined under a fluorescent microscope (ZEISS Apotome 2; Germany). TUNEL and PI-positive nuclei/cells were analyzed at excitation wavelengths in the range of 450–500 nm and 500–550 nm respectively.

### Sciatic nerve in situ efferocytosis

Sciatic nerve IF staining was performed as described in our previous studies [10, 11] with deparaffinization and rehydration as above. Antigen retrieval was performed using 10 mM sodium citrate buffer (pH6.0) for 20 min at 95 °C. Permeabilization and blocking of nonspecific binding were performed using 1% Triton X-100 and 5% goat serum respectively. Primary antibody staining was performed with anti-CD206 (1:400; #141703; BioLegend) and anti-MP0 (1:1000; #PZ0; Aves Labs), and followed by incubation with the appropriate secondary antibody: Alexa Fluor 488 (1:1000; #A11008; Invitrogen) and Alexa Fluor 647 (1:1000; #A21449; Invitrogen). Staining without primary antibodies was used as a control for nonspecific fluorescence. Nuclei were counter-stained using ProLong^TM^ Gold anti-fade reagent with DAPI and sections were examined under a fluorescent microscope. Imaged nerve tissues were analyzed using NIH-ImageJ software, for the quantification of myelin content.

### Isolation, culture, and characterization of mouse bone marrow-derived macrophage

Mouse bone marrow-derived macrophage (BMMØ) isolation and ex vivo expansion was performed as previously described with slight modifications [14]. Femur and tibial bone marrow cavities were flushed, resuspended in the DMEM complete medium [(DMEM supplemented with 10% (vol/vol) FBS, 1% (vol/vol) streptomycin/penicillin and 50% (vol/vol) and L929 conditioned medium (as a macrophage colony-stimulating factor (M-CSF))] was passed through a 70-μm-cell strainer and then incubated in a humidified chamber at 37 °C with 5% CO_2_. After differentiation for 5–6 days, cells were subcultured (passage 1), maintained, and used for flow cytometry analysis using CDD11b-APC (1:100; #553312; BD Biosciences) and F4/80-PE (1:100; #565410; BD Biosciences).

### Macrophage cell viability assay

Cell viability tests were conducted as per kit instructions (MTT [(3-(4,5-dimethylthiazol-2-yl)−2,5-diphenyltetrazolium bromide)]) with slight modifications (#11465007001; Roche). Briefly, BMMØs were seeded at a concentration of 2 × 10^4^ cells/well in a 96 well plate and incubated for 24 h at 37 °C and 5% CO_2_. Cells were treated with EPO at a concentration of 0.1, 0.25, 1, 5, 10, 25, and 50 IU/mL for 24 and 72 h. After treatment, the MTT reagent was added to a final concentration of 0.5 mg/mL of total volume and incubated for 3 h in the 5% CO_2_ incubator. After incubation, the MTT reagent was removed and solubilization solution dimethylsulfoxide (DMSO) (#4-X, ATCC) was added to each well and stirred using a gyratory shaker to enhance the dissolution of formazan crystals. The absorbance was recorded to calculate cell viability percentages using a microplate spectrophotometer (SPECTRAmax^®^ 340PC) at 570 nm using 630 nm as a reference wavelength. Untreated cells were used as controls.

### Induction of apoptosis in bone marrow-derived macrophages

The induction of apoptosis in BMMØs was conducted using lipopolysaccharide (LPS) (#L4516; Sigma-Aldrich) at 50 ng/mL for 24 h. Apoptosis (%) was analyzed by flow cytometry (BD Biosciences LSR Fortessa 16) using Annexin V-FITC and 7AAD kit (# 559763; BD Biosciences).

### Flow cytometry analysis

Single-cell suspensions of the LPS/ EPO-treated macrophages (BMMØs and PMØs) and peritoneal lavage cells generated from the mice were washed twice in 1XDPBS and resuspended in 1X flow cytometry staining buffer (#FC001; R&D) according to manufacture instructions before labeling with either antibodies or dyes depending on the experiment. After incubation, cells were washed in 1X FACS buffer and analyzed immediately by flow cytometry, and data were collected with BD FACSDiva™ v7 software and were evaluated using FlowJo™ v10.8 Software.

### RNA extraction and qRT-PCR analysis

Total RNA was extracted from the BMMØs (Groups: Control-no treatment, LPS-50ng/mL and LPS + EPO-50 ng/mL + 5 IU/mL). LPS treatment was for 24 h, whereas EPO treatment lasted 72 h. The miRNeasy mini kit (#217004; Qiagen) was used and RNA was reverse transcribed to cDNA using iScript^TM^ reverse transcription supermix (#1708840; Bio-Rad), according to the manufacturer’s instructions. Primers were purchased from Invitrogen (Life Technologies) and the sequences are listed in Supplemental Table S1. qRT-PCR was performed using Fast SYBR Green Master Mix (#4385612; Applied Biosystems) with a Step One Plus Real-Time PCR System (Applied Biosystems; California, USA) for detection of gene expression. Relative mRNA expression of the target genes was normalized to glyceraldehyde 3-phosphate dehydrogenase (GAPDH) gene expression and data were represented as fold change versus the respective control. In parallel, the RNA integrity number (RIN) for the standardization of RNA quality was performed by Agilent Bioanalyzer 2100 (Agilent Technologies; Germany).

### Isolation, culture, and characterization of mouse sciatic nerve Schwann cells

Mouse sciatic nerve derived Schwann-cell (SNSC) isolation and ex vivo expansion was performed as described in previous studies with slight modifications [15–17]. Briefly, mice were euthanized using isoflurane followed by cervical dislocation, and skin was disinfected using 70% alcohol. Then, under the microscope, a lateral skin incision (≈3 cm) was made along the length of the femur and the sciatic nerve was exposed to harvest 1.5 cm of nerve, which was placed in a Petri dish containing an ice-cold DMEM basal medium (#11995073; Gibco™). The epineurium layer was removed with forceps and nerve fibers were transferred to a new dish to avoid fibroblast contamination. Mechanical teasing apart of nerve fibers was continued until fine breakdown and then nerve tissue was subjected to enzymatic dissociation [0.2% collagenase type I (#17018029; Gibco™): 0.2% dispase II (#D4693; Millipore Sigma) in a DMEM basal media] with intermittent (every 10 min) mechanical interruption by pipetting for 50 min. Enzymatic activity was stopped with 10% fetal bovine serum (#30-2020; ATCC) and the cell suspension was filtered using a 100-μm-cell strainer, centrifuged at 300 × *g* for 5 min, and resuspended in a DMEM medium (DMEM supplemented with 10% (vol/vol) FBS, 1% (vol/vol) streptomycin/penicillin). Finally, cells were plated in a PLL (poly-l-lysine) (#P4707; Millipore Sigma) coated T25-flask and were incubated in a humidified chamber at 37 °C with 5% CO_2_. After overnight incubation cultured cells were supplemented with 10 μM cytosine β-d-arabinofuranoside hydrochloride (#C6645; Millipore Sigma) for 24 h to kill fibroblast cells that actively divide. Cells were then incubated with mouse Schwann-cell medium [(#M1700-57; ScienCell^TM^; supplemented with 5% (vol/vol) FBS, 1% (vol/vol) streptomycin/penicillin, and 1% (vol/vol) Schwann cells growth factor)], which was changed every 2–3 days. After 8–10 days, cells were passaged (passage 1), maintained, and used for IF analysis using S100 (1:100; #MA5-12969; Invitrogen), p75NTR (1:250; #AB1554; Sigma-Aldrich), and MPZ (1:1000; #PZO; AvesLab) followed by incubation with the appropriate secondary antibody Alexa Fluor 594 (1:500; #A11032; Invitrogen), Alexa Fluor 488 (1:500; #A11008; Invitrogen), and Alexa Fluor 647 (1:500; #A21449; Invitrogen).

### Induction of apoptosis in sciatic nerve derived Schwann cells

To conduct macrophage efferocytosis studies, we induced apoptosis of SNSCs using 250 μM hydrogen peroxide (H_2_O_2_) (#H325-100; Fisher Scientific) overnight (16 h). After induction, apoptosis was analyzed by flow cytometry using Annexin V-FITC and 7AAD kit.

### Isolation, culture, and characterization of mouse peritoneal macrophages

Mouse peritoneal-derived macrophage (PMØ) isolation and ex vivo expansion was performed as previously described with slight modification [18]. Briefly, mice were euthanized using isoflurane followed by cervical dislocation. Next, abdominal skin was disinfected using 70% alcohol and 10 ml ice-cold PBS was injected IP and peritoneal fluid was aspirated (8 min later), centrifuged at 300 × *g* for 5 min, resuspended in DMEM complete medium (see BMMØs isolation protocol), seeded, and incubated in a humidified chamber at 37 °C with 5% CO_2_. After 5–6 days, cells were subcultured (passage 1), maintained, and used for cellular characterization by flow cytometry using CD11b-APC (#553312; BD Biosciences) and F4/80-BV421 (#565411; BD Biosciences).

### In vitro macrophage efferocytosis

The in vitro efferocytosis studies were performed using both BMMØs and PMØs, and apoptotic SNSCs as previously described with slight modifications [19]. Briefly, macrophages were seeded into four-well slides at 2 × 10^4^ cells per well and incubated overnight in the humidified chamber at 37 °C and 5% CO_2_. Macrophages were treated with either LPS (50 ng/mL), or LPS (50 ng/mL) + EPO (5 IU/mL), or not treated (control group) for 24 h and then incubated with PKH26 (cell membrane labeling dye; # MINI26; Sigma-Aldrich) labeled apoptotic SNSCs for 3 h (ratio: 1 macrophage:2 SNCSs). After incubation, macrophages were washed (1XDPBS) and labeled with Flash Phalloidin Green 488 (1:100; #42420; Biolegend) for intracellular cytoskeleton F-actin and coverslips were mounted on glass slides with ProLong^TM^ Gold anti-fade reagent with DAPI for examination under a fluorescent microscope. Efferocytosis percentages were calculated using NIH-ImageJ Version 1.53 software (USA) by analyzing the ratio of PKH26 to DAPI.

### In vivo peritoneal macrophage phagocytosis

PKH26 labeling of apoptotic SNSCs was performed as per manufacturer instructions (#MIDI26; Sigma-Aldrich). PKH26-labeled apoptotic SNSCs (~5 × 10^6^ cells) were injected into the peritoneal cavities of mice (*n* = 3 animals/group) 3 days after beginning IP EPO (5000 IU/kg) or saline (normal saline, 0.1 ml/mouse) dosing. Two hours later, mice were sacrificed and peritoneal lavage was performed as above to recover PMØs, which were resuspended in 1X flow cytometry staining buffer and incubated with CD11b-APC (1:100; #553312; BD Biosciences), CD206-FITC (1:100; #141703; BioLegend), and F4/80-PE (1:100; #565410; BD Biosciences) antibodies at 4 °C for 30 min. Efferocytosis percentages (PKH26 positivity in CD11b + CD206^+^ cells) and macrophage infiltration (CD11b + F4/80^+^ cells) were determined using flow cytometry.

### Statistical analyses

All data were analyzed using GraphPad Prism Version 8.4.3 (San Diego, USA). Comparisons between three groups with n≥3 were performed via ordinary one-way analysis of variance (ANOVA), and Tukey’s multiple comparisons test after confirmation of normally distributed data. For comparisons of two groups, (*n* per group ≥3), two-tailed, unpaired *t*-tests were used. All values are presented as means ± SEM. Significance levels (*P* values <0.05) were documented using standard symbols (*, **, ***, **** and #, ##, ###, #### correspond to *P* < 0.05, *P* < 0.0021, *P* < 0.0002, and *P* < 0.0001, respectively).

## Results

### EPO treatment attenuates SN apoptosis following SNCI

**Fig. 1:**
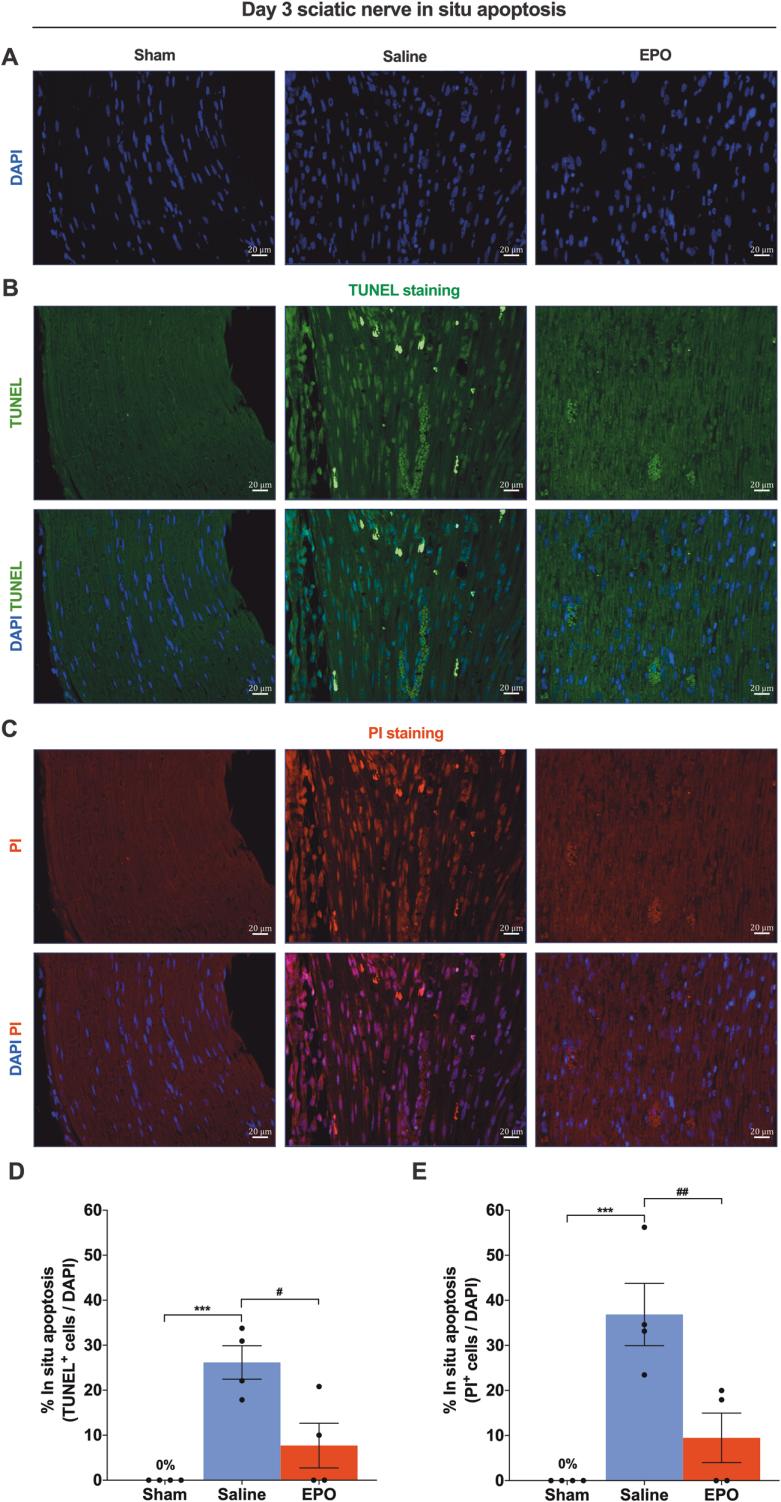
On day 3, EPO effectively attenuates SN in situ apoptosis following SNCI. **A** DAPI staining shows cellular infiltration of SN. **B**, **C** Representative IF images of TUNEL and PI staining of SN shows that EPO treatment (5000 IU/kg/IP, immediately after surgery and on post-surgery day 1 and 2) significantly attenuates apoptosis as compared to saline treatment (normal saline, 0.1 ml/IP/mouse) and the result of the percentage of in situ apoptosis (TUNEL and PI^+^/DAPI) are mentioned in the bar graph (**D**, **E**). SN of Sham animals shows zero percentage of apoptosis. Each image represents six images from four different SN, a total of 24 images/group. Scale bar, 20 μm; magnification, 40x. One-way ANOVA, Tukey’s multiple comparisons test. Data were expressed as means ± SEM, ***P < 0.0002 vs. sham, #*P* < 0.05 and ##*P* < 0.0021 vs. Saline, *n* = 4/group.

**Fig. 2:**
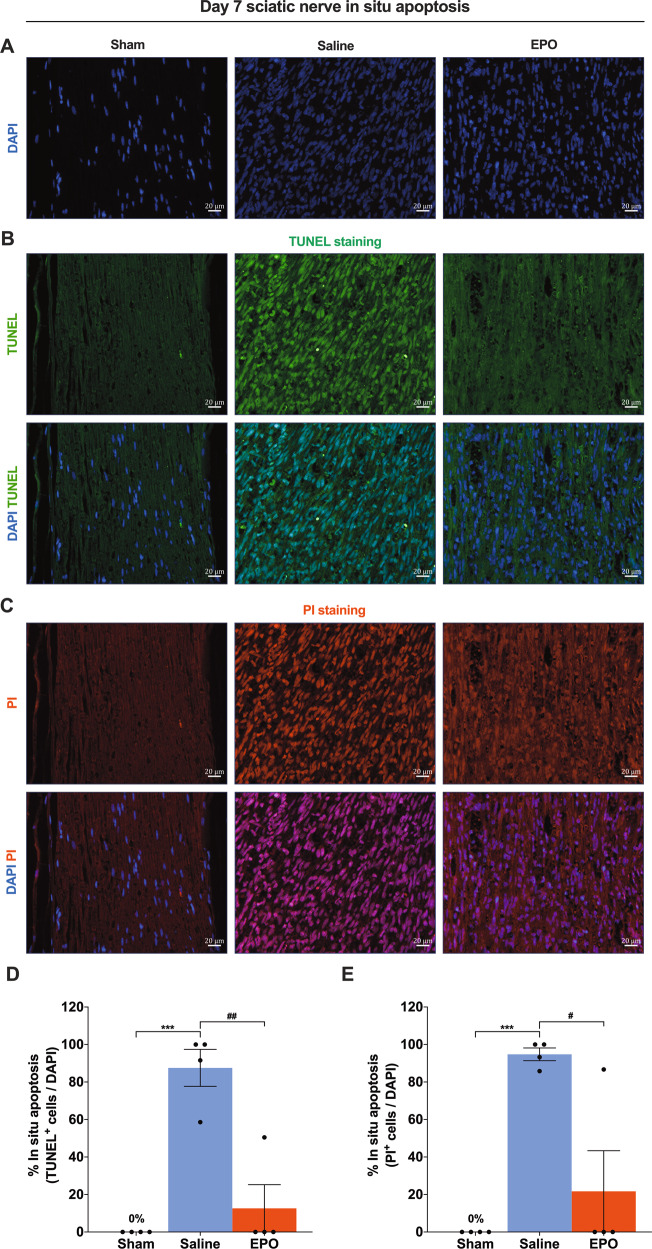
On day 7, EPO significantly attenuates SN in situ apoptosis following SNCI. **A** DAPI staining shows cellular infiltration of SN. **B**, **C** Representative IF images of TUNEL and PI staining of SN shows that EPO treatment (5000 IU/kg/IP, immediately after surgery and on post-surgery day 1 and 2) significantly attenuates apoptosis as compared to saline treatment (normal saline, 0.1 ml/IP/mouse) and the result of the percentage of in situ apoptosis (TUNEL and PI^+^/DAPI) are mentioned in the bar graph (**D**, **E**). SN of Sham animals shows zero percentage of apoptosis. Each image represents six images from four different SN, a total of 24 images/group. Scale bar, 20 μm; magnification, 40x. One-way ANOVA, Tukey’s multiple comparisons test. Data were expressed as means ± SEM, ***P < 0.0002 vs. sham, #*P* < 0.05, and ##*P* < 0.0021 vs. Saline, *n* = 4/group.

An injury to the SN initiates a sequence of cellular and molecular changes that often result in enduring functional deficits [20, 21]. Among these changes, SC apoptosis is a significant pathological feature fundamental for nerve regeneration and functional improvement [22, 23]. To determine the functional effects of EPO on neuronal apoptosis following SNCI, we co-stained SN using TUNEL and PI. On day 3, EPO treatment significantly protected against apoptosis compared to saline treatment (7.70% ± 4.29 vs. 26.16% ± 3.22 and 9.48% ± 4.75 vs. 36.85% ± 5.98) (Fig. 1B–D, C–E; #*P* < 0.05, ##*P* < 0.0021). By day 7, EPO treatment continued to show protective effects (vs. day 3 EPO) against apoptosis (12.61% ± 10.92 and 21.66% ± 18.76 vs. 7.70% ± 4.29 and 9.48% ± 4.75), in comparison to a large increase in apoptosis in saline-treated mice (87.53% ± 8.53 and 94.76% ± 2.92) (Figs. 2B–D, C–E vs. 1B–D, C–E; ***P < 0.0002, #*P* < 0.05, ##*P* < 0.0021). Ipsilateral SN of sham mice had no (0%) apoptosis on day 3 or day 7 (Figs. 1B–E, 2B–E). Even after injury, the amount of apoptosis in EPO-treated mice was not significantly greater than in sham mice, in comparison with saline-treated mice, which had significantly greater apoptosis (Figs. 1B–E,2B–E; ***P < 0.0002). Figs. 1A, 2A show total nuclei (DAPI^+^) in the sciatic nerve used to assess apoptosis (vs. TUNNEL^+^/PI^+^) following SNCI. EPO had a significant effect on preventing apoptosis, which may be the basis for the promotion of functional improvement after injury.

### EPO treatment promotes M2 phenotype macrophage phagocytosis and augments SN function following SNCI

**Fig. 3:**
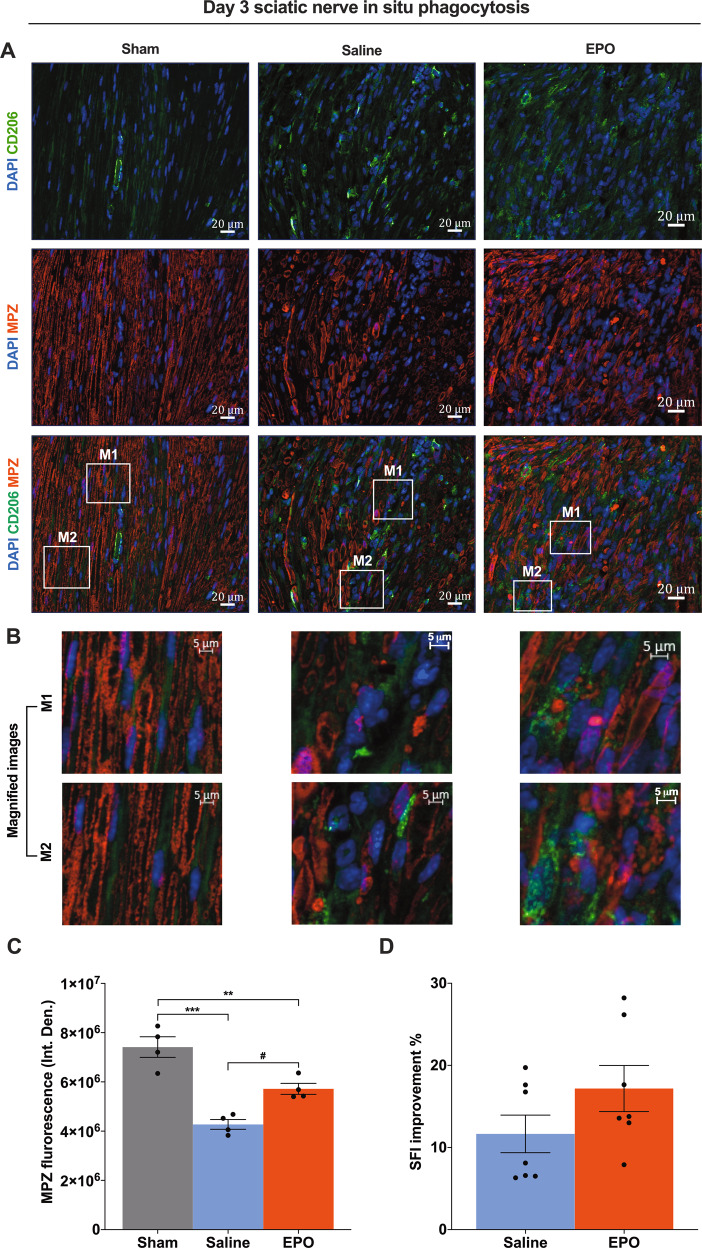
On day 3, EPO effectively activates CD206^+^ macrophage phagocytosis of myelin debris, which protects myelin breakdown, and improves functional recovery following SNCI. **A** Representative IF images of CD206 (M2 phenotype macrophages) and MPZ (Myelin) staining of SN shows that EPO treatment (5000 IU/kg/IP, immediately after surgery and on post-surgery day 1 and 2) effectively control phagocytosis of myelin debris and which significantly protects myelin breakdown as compared to saline treatment (normal saline, 0.1 ml/IP/mouse) and are depicted in magnified images (**B**) and the results of the integrated density of MPZ are depicted in the bar graph (**C**). M1 and M2 are random subset images of the SN section. **D** EPO treatment effectively improves %SFI. Each image represents six images from four different SN, a total of 24 images/group. Scale bar, 20 μm; magnification, 40x. One-way ANOVA, Tukey’s multiple comparisons test. Data were expressed as means ± SEM, ***P < 0.0002, Saline vs. sham; **P < 0.0021, EPO vs. sham, #*P* < 0.05, Saline vs. EPO, *n* = 4/group (CD206/MPZ staining analyses). Unpaired *t*-tests, *n* = 7/group (SFI analyses).

**Fig. 4:**
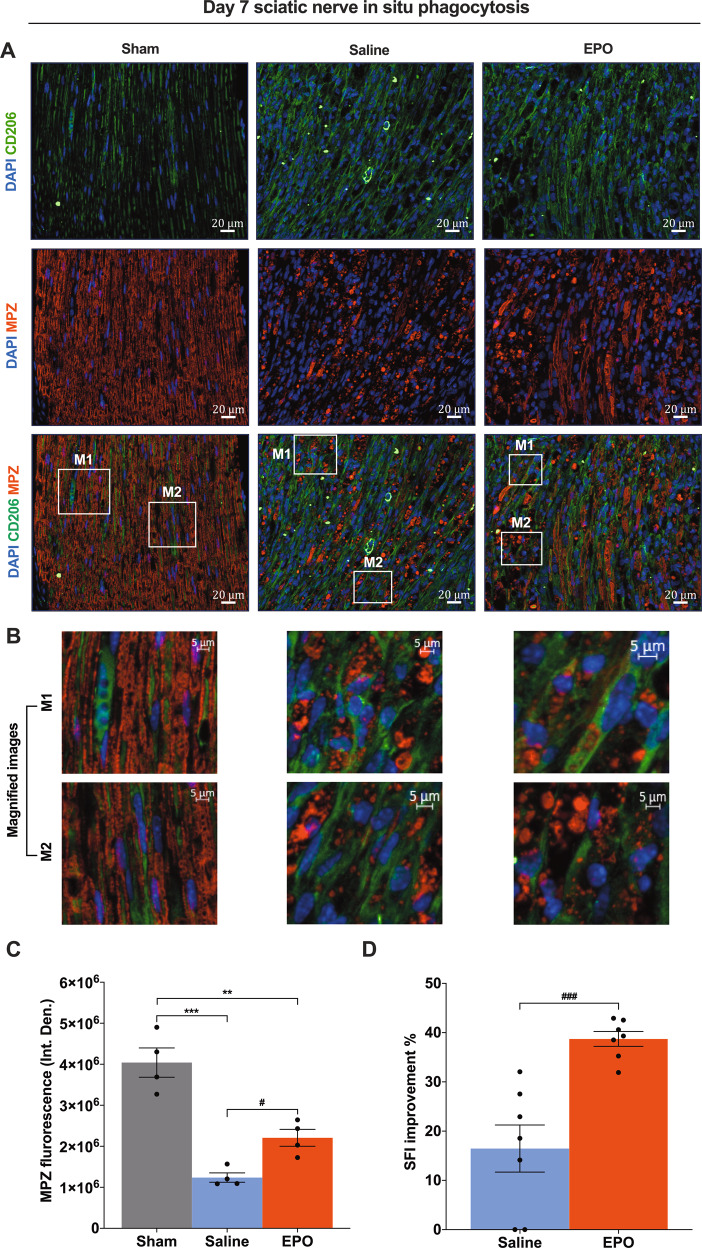
On day 7, EPO significantly activates CD206^+^ macrophage phagocytosis of myelin debris, which improves myelination and functional recovery following SNCI. **A** Representative IF images of CD206 (M2 phenotype macrophages) and MPZ (Myelin) staining of SN shows that EPO treatment (5000 IU/kg/IP, immediately after surgery and on post-surgery day 1 and 2) effectively control phagocytosis of myelin debris and which significantly improves myelination as compared to saline treatment (normal saline, 0.1 ml/IP/mouse) and are depicted in magnified images (**B**) and the results of the integrated density of MPZ are depicted in the bar graph (**C**). M1 and M2 are random subset images of the SN section. **D** EPO treatment significantly improves %SFI. Each image represents six images from four different SN, a total of 24 images/group. Scale bar, 20 μm; magnification, 40x. One-way ANOVA, Tukey’s multiple comparisons test. Data were expressed as means ± SEM, ***P < 0.0002, Saline vs. sham; **P < 0.0021, EPO vs. sham; #*P* < 0.05, EPO vs, saline, *n* = 4/group (CD206/MPZ staining analyses). Unpaired *t*-tests, ###*P* < 0.0002, EPO vs, saline, *n* = 7/group (SFI analyses).

An ideal therapeutic strategy can promote a balance between clearance of SC debris and remyelination following nerve trauma [24, 25]. Macrophage recruitment and activation play a vital role in the recognition and clearance of dying and dead cells as well as the resolution of the inflammatory milieu to support regeneration and tissue repair processes. To demonstrate whether EPO-activated M2 phenotype macrophages (CD206^+^) protect and augment myelination through phagocytosis of myelin debris, we imaged the SN section for the presence of myelin debris (MPZ positivity) within the cytoplasm of CD206^+^ macrophages on day 3 and day 7 post-SNCI. Figs. 3A, 4A show macrophage phagocytosis via engulfing myelin debris with either EPO or saline treatment (magnified in Figs. 3B, 4B). At these early post-injury time points, EPO treatment significantly attenuates myelin breakdown (Fig. 3A–C; #*P* < 0.05) and augments remyelination (Fig. 4A–C; #*P* < 0.05) compared to saline treatment. In parallel evaluated motor function in identically injured and treated animals and found that within a week post-injury, EPO treatment resulted in significant functional improvement in walking compared to saline (SFI: 38.72% ± 1.39 vs. 16.46% ± 4.42), (Fig. 4D; ###*P* < 0.0002). This effect was even evident as early as day 3 (17.18% ± 2.59 vs. 11.66% ± 2.11) (Fig. 3D)—a time point well before canonical descriptions of neuronal regeneration can occur. Our findings support the idea that EPO-mediated functional improvement is occurring at the same time points where changes in macrophage phagocytosis can be documented inside the injured nerve ((i.e., enhanced anti-apoptosis (Figs. 1, 2), M2 macrophage phenotype activation, phagocytosis, and myelination (Figs. 3, 4)).

### EPO treatment promotes macrophages phenotype transition following LPS treatment

Macrophages play a central role following peripheral nerve injuries that is related to the phenotypic transition from M1(pro-inflammatory) to M2 (anti-inflammatory) and is fundamental to tissue homeostasis and nerve regeneration [26, 27]. We aimed to delineate the functional role of EPO on the phenotypic transition of BMMØs during the resolution of endotoxin-induced inflammation.

We first characterized BMMØs and determined the optimal concentrations of EPO and endotoxin (LPS) to ensure that these concentrations corresponded to those expected in tissues after injury and treatment. Cell-surface marker analyses using flow cytometry (CD11b and F4/80 positivity) confirmed a high purity of BMMØs (99.70% ± 0.16) (Supplemental Fig. S1) and MTT assays showed the percentage of those cells that remained viable at various EPO concentrations selected based on human plasma EPO levels [28, 29] (0.1, 0.25, 1, 5, 10, 25, and 50 IU/mL) at both 24 and 72 h. EPO treatment in a range from 0.1–50 IU/mL was not toxic to incubated BMMØs at both 24 and 72 h (Supplemental Fig. S2A, B), and EPO supported cell viability at all but the highest and lowest concentrations tested (Supplemental Fig. S2A at 24 h and Supplemental Fig. S2B at 72 h). Using this indirect measure of cell metabolic activity, we selected an ideal concentration of EPO to use in vitro that corresponded well to human treatments. Next, we treated BMMØs with varying doses of EPO (5, 25, and 50 IU/mL) in the presence of concentrations of LPS (50 and 100 ng/mL) for 24 h and analyzed the amount of apoptosis by flow cytometry (early apoptosis (Annexin V) vs. late apoptosis (7AAD) positivity, gating strategy Supplemental Fig. S3A) and found that both LPS concentrations significantly increased early and late apoptosis (Supplemental Fig. S3B), which was effectively attenuated by EPO (5, 25, and 50 IU/mL) (Supplemental Fig. S3B). We selected 5 IU/mL of EPO as an effective therapeutic dose and 50 ng/mL of LPS for the remainder of in vitro cell-culture experiments.

**Fig. 5:**
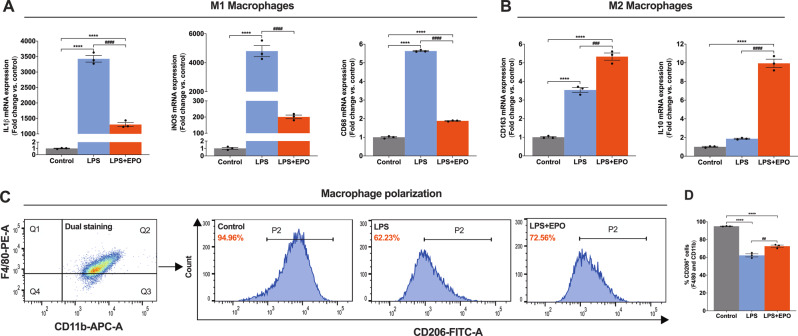
EPO treatment promotes BMMØs phenotype transition following LPS treatment. qRT-PCR data shows that EPO treatment significantly attenuates pro-inflammatory genes (IL1β, iNOS, and CD68; M1 macrophage markers) (**A**) and upregulates anti-inflammatory genes (IL10 and CD163; M2 macrophage markers) (**B**) following LPS treatment. BMMØs treated with LPS (50 ng/mL) for 24 h and EPO (5 IU/mL) for 72 h. (**C**). Flow cytometry gating strategy of dual staining BMMØs (CD11b + F4/80), where that shows (P2, histograms) LPS + EPO treatment significantly shifts expression of CD206^+^ M2 phenotype macrophages as compared to LPS treatment, and the results are represented as a bar graph (**D**). One-way ANOVA, Tukey’s multiple comparisons test. Data were expressed as means ± SEM, ****P < 0.0001, Control vs. LPS and LPS + EPO; ###*P* < 0.0002, and ####*P* < 0.0001, LPS vs. LPS + EPO, *n* = 3.

Next, we treated BMMØs with either LPS (50 ng/mL) alone or in conjunction with EPO (5 IU/mL), and compared results to a no-treatment control group. We assessed the quality of extracted total RNA using RNA integrity number (RIN) (Supplemental Fig. S4A, B) to protect against the possibility of measuring nonspecific changes in gene expression. We then used qRT-PCR to analyze cultures for the expression of genes associated with either the M1 macrophage phenotype (IL1β, iNOS, and CD68) or the M2 macrophage phenotype (CD163 and IL10) and found that EPO significantly attenuated LPS-induced pro-inflammatory genes expression (IL1β, iNOS, and CD68) that were significantly upregulated with LPS alone (Fig. 5A; ####*P* < 0.0001). When compared to the control (untreated, and non-LPS stressed) cultures, IL1β and CD68 were significantly increased with LPS-alone and this increase was significantly blunted with the addition of EPO (Fig. 5A; ****P < 0.0001). This was also true with iNOS gene expression where EPO treatment levels were not significantly different than controls (Fig. 5A). Notably, CD163 and IL10 levels were significantly greater in LPS cultures treated with EPO compared with LPS alone (Fig. 5B; ###*P* < 0.0002 and ####*P* < 0.0001), and in the case of IL10, LPS-alone could not increase levels significantly above control without the addition of EPO (Fig. 5B). We further conducted flow cytometry analyses to verify our above result and found that LPS significantly decreased the macrophage M2 cell-surface marker CD206 compared to control (Fig. 5C, D; ****P < 0.0001), and that the addition of EPO significantly increased CD206 expression (Fig. 5C, D; ##*P* < 0.0021). EPO increases the expression of genes and markers that are related to phagocytosis in macrophages.

### EPO treatment prevents M2 phenotype macrophage apoptosis after LPS induction

**Fig. 6:**
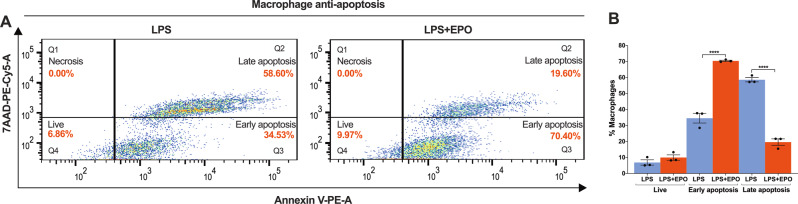
EPO treatment augments M2 phenotype macrophage anti-apoptosis function following LPS treatment. **A** Flow cytometry data shows that LPS + EPO treatment significantly attenuates % late apoptosis (Q2 quadrant) and as a result, it increases the percentage of early apoptosis (Q3 quadrant) and the live cells (Q4 quadrant) as compared to LPS treatment. None of the treatments shows positive for necrosis. BMMØs treated with LPS (50 ng/mL) for 24 h and EPO (5 IU/mL) for 72 h. **B** The results of apoptosis are mentioned in the bar graph. One-way ANOVA, Tukey’s multiple comparisons test. Data were expressed as means ± SEM, ****P < 0.0001, LPS vs. LPS + EPO, *n* = 3.

In the setting of nerve injury, macrophages themselves can become apoptotic from the effects of oxidative stress and inflammatory cytotoxins [30, 31]. To determine the effect of EPO on apoptosis of M2 phenotype macrophages, we treated BMMØs with LPS (50 ng/mL) with and without EPO (5 IU/mL) for 72 h and analyzed apoptosis as measured by Annexin V/7AAD positivity using flow cytometry. In comparison with LPS exposure alone, EPO treatment significantly attenuated late, irreversible apoptosis (19.60% ± 1.67 vs. 58.60% ± 1.10) and increased the relative population of cells in early, reversible apoptosis (70.40% ± 0.33 vs. 34.53% ± 2.46). EPO also increased the number of live, non-apoptotic cells (9.97% ± 1.35 vs. 6.86% ± 1.36) (Fig. 6A, B; ****P < 0.0001). EPO’s overall effect in these conditions was to shift BMMØs away from committed apoptotic cell death.

### EPO treatment supports M2 phenotype macrophage efferocytosis of apoptotic SNSCs, in vitro

The clearance of damaged SCs by professional phagocytes, macrophages is a critical element of the recovery from peripheral nerve trauma [3, 32]. We measured the effect of EPO on both BMMØs and PMØs engaged in efferocytosis of apoptotic SNSCs in vitro to gauge the effect of this fundamental element of macrophage function.

We first characterized the induction of apoptosis of SNSCs, confirming the identity and purity of SCs derived from the sciatic nerve using three markers (S100, p75NTR, and MPZ) by IF staining (Supplemental Fig. S5). We next confirmed the ability to quantify the percentage of SNSCs undergoing apoptosis during induction with H_2_O_2_ using IF for Annexin V positivity (Annexin V positive: 97.52% ± 0.83 vs. negative: 2.47% ± 0.83) (Supplemental Fig. S6A, B). Flow cytometry analysis confirmed that SNSCs are less susceptible to apoptosis from LPS exposure at a range of concentrations (50–500 ng/ml) (Supplemental Fig. S7A, B) than overnight induction with 250 μM H_2_O_2_ (both Annexin V and 7 AAD positivity: 92.86% ± 0.49, Annexin V alone (early apoptosis): 7.13% ± 0.50, double negative: 0%) (Supplemental Fig. S6C, D). Next, using cell-surface marker analyses (CD11b and F4/80 positivity) by flow cytometry confirmed a high purity of PMØs (99.26% ± 0.07) (Supplemental Fig. S8).

**Fig. 7:**
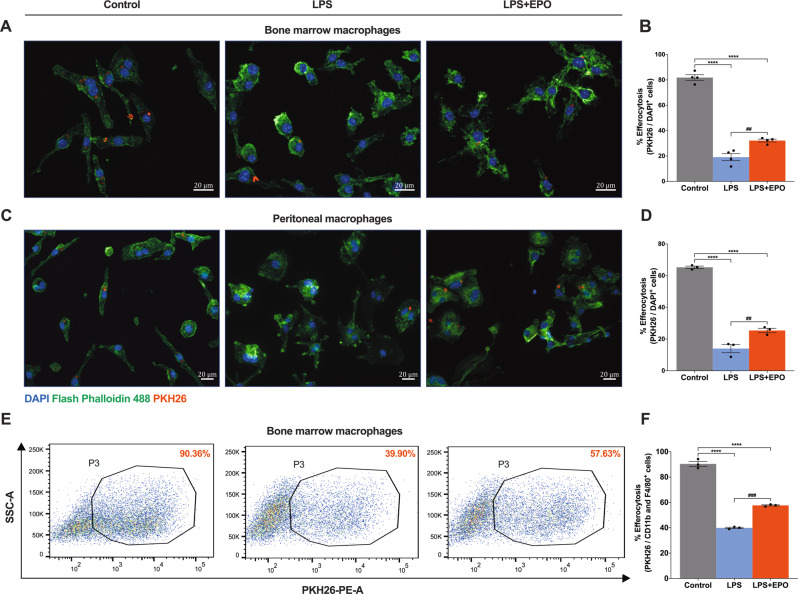
EPO treatment enhances M2 phenotype macrophage efferocytosis of apoptotic SNSCs, ex vivo. IF staining of BMMØs (**A**) and PMØs (**C**) (Flash phalloidin, green) shows that LPS + EPO treatment significantly enhances efferocytosis of apoptotic SNSCs (PKH26, red) as compared to LPS treatment and the results are mentioned in the bar graph (**B**, **D**). In the BMMØs study, each image represents five images from four independent experiments, a total of 20 images/group. In the PMØs study, each image represents five images from three independent experiments, a total of 15 images/group. The percent efferocytosis was calculated using PKH26 positivity per cell (DAPI). Scale bar, 20 μm; magnification, 40x. One-way ANOVA, Tukey’s multiple comparisons test. Data were expressed as means ± SEM, ****P < 0.0001, Control vs. LPS and LPS + EPO; ##*P* < 0.0021, LPS vs. LPS + EPO, *n* = 4 (BMMØs study), *n* = 3 (PMØs study). **E** Flow cytometry data show that LPS + EPO treatment significantly augments %efferocytosis of apoptotic SNSCs as compared to LPS treatment and the results are mentioned in the bar graph (**F**). One-way ANOVA, Tukey’s multiple comparisons test. Data were expressed as means ± SEM, ****P < 0.0001, Control vs. LPS and LPS + EPO; ###*P* < 0.0002, LPS vs. LPS + EPO, *n* = 3.

We exposed BMMØs cultures in three conditions (control untreated, LPS, and LPS + EPO) to the above prepared apoptotic SNSCs. The inclusion of EPO treatment in BMMØ cultures significantly increased efferocytosis over LPS treatment alone (32.15% ± 0.97 vs. 19.08% ± 2.45) (Fig. 7A, B; ##*P* < 0.0021) but not to the level of efferocytosis seen in the un-stressed control BMMØ cultures (Fig. 7A, B; ****P < 0.0001). We performed the same experiment with macrophages derived from the peritoneum PMØs to see if these effects were independent of the macrophage source and obtained similar results (25.41% ± 1.15 vs. 13.99% ± 2.13) (Fig. 7C, D; ##*P* < 0.0021) as with BMMØs. This was further confirmed using flow cytometry (see Supplemental Fig. S9A, B for gating strategy), where the addition of EPO to LPS increased BMMØs efferocytosis compared to LPS-alone (57.63% ± 0.37 vs. 39.90% ± 0.40) (Fig. 7E, F; ###*P* < 0.0002). The gating strategy of EPO potentiates the capacity for macrophages to engulf dead and dying SNSCs in vitro.

### EPO treatment augments M2 phenotype peritoneal macrophage phagocytosis, in vivo

**Fig. 8:**
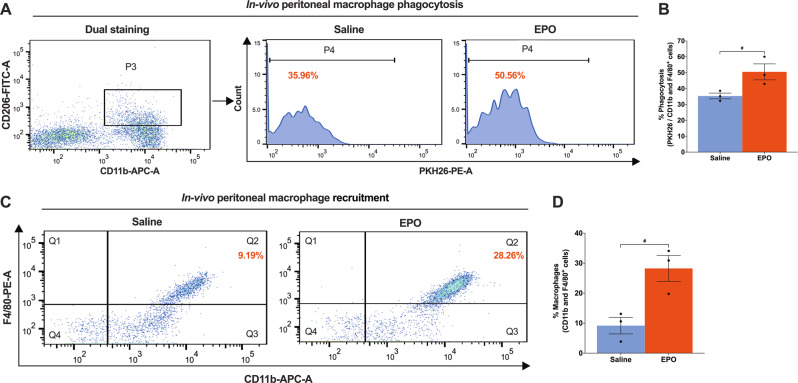
EPO treatment augments M2 phenotype peritoneal macrophage phagocytosis, in vivo. **A** Flow cytometry gating strategy of dual staining (CD11b + CD206^+^), where that shows (P4, histogram) EPO treatment (5000 IU/kg/IP, immediately after surgery and on post-surgery day 1 and 2) significantly augments percent phagocytosis of intraperitoneally injected apoptotic SNSCs (PKH26 labeled) as compared to saline treatment (normal saline, 0.1 ml/IP/mouse) and the results are denoted in the bar graph (**B**). **C** Flow cytometry data shows EPO significantly recruits PMØs (CD11b + F4/80^+^) following intraperitoneal injection of apoptotic SNSCs (PKH26 labeled) as compared to saline treatment and the results are denoted in the bar graph (**D**). Unpaired *t*-tests. Data were expressed as means ± SEM, #*P* < 0.05, Saline vs. EPO, *n* = 3/group.

The peritoneal compartment in the mouse presents an alternative, in vivo venue to test the capacity of macrophages to clear SNSC debris. To see if administration of EPO increases phagocytosis of M2 phenotype peritoneal macrophages (CD206 + CD11b^+^), we injected PKH26-labeled apoptotic Schwann cells (~5 × 10^6^ cells/mouse) intraperitoneally in mice either pretreated with saline or EPO (5000IU/kg/day, IP for 3 days). Two hours after injection of apoptotic SNSCs, mice were sacrificed and peritoneal lavage samples were assessed for PMØ phagocytosis of debris for flow cytometry of CD206^+^ macrophages. EPO treatment significantly increased engulfment activity in PMØ samples over saline-treated mice (50.56% ± 4.10 vs. 35.36% ± 1.48) (Fig. 8A, B; #*P* < 0.05), but not the percentage of CD206^+^ PMØs (9.97% ± 1.28 vs. 8.93 ± 1.15) (Supplemental Fig. S10). Interestingly, the recruitment of M1 phenotype macrophages (F4/80 + CD11b^+^) was significantly affected by EPO treatment in vivo after following injection of apoptotic SNSCs over saline-treated mice (28.26% ± 3.53 vs. 9.19 ± 2.25) (Fig. 8C, D; #*P* < 0.05). At just 2 h post-exposure to apoptotic SNSCs, EPO may regulate both the M1 and M2 macrophage phenotypes to allow early recruitment of M1 macrophages, as well as early phenotypic transitions to M2 phenotype macrophages in the setting of SC clearance. This may have direct relevance to post-SNCI functional recovery.

## Discussion

Inflammation, apoptosis, and removal of dying neuronal cells are the primary pathophysiological consequences of nerve injury [33, 34]. Infiltrating immune cells play a critical role post-SNCI and inflammation itself undergoes a transition from an initial pro-inflammatory reaction to an anti-inflammatory phase. This is critical for nerve repair and structural modification [35, 36]. However, a persistent and severe pro-inflammatory reaction can impair peripheral nerve recovery, which depends on macrophages to clear dead cells in advance of SC orchestration of nerve regeneration [2, 37]. EPO signaling seems to have a role in guiding this transition. EPO is able to turn off some effects of inflammation [10, 38], and may exert control through macrophage inflammation and efferocytosis [39]. The significance of the effects of EPO on debris clearance in the nerve after the injury is unknown, despite our work and the work of others motivating clinical translation of EPO for TPNI [40, 41]. Since the debris to be cleared after TPNI involves apoptotic SCs and the prevention of secondary necrosis as well as further tissue damage [42, 43], we hypothesized that EPO may affect this critical clearance activity in M2 macrophages.

Our data shows that EPO treatment significantly attenuates apoptosis within the nerve after SNCI and activates the phagocytic function of M2 macrophages to clear SC debris. This, in turn, protects myelin and promotes functional recovery. M2 phenotype macrophage phagocytosis in the injured peripheral nerve is key to clearance of debris, and EPO treatment mice suffer significantly less apoptosis on early days after SNCI than saline-treated control mice through the activation of CD206^+^ M2 macrophage phagocytosis of myelin debris early after injury. This results in less myelin destruction and more remyelination within a week of SNCI—well before axonal regeneration is expected to occur. Here we present in vivo and in vitro evidence that these events, including efferocytosis and apoptosis prevention, are sensitive to EPO treatment in support of the findings of others who investigated EPO in other disease processes [6, 7, 44–46].

Although EPO is well known for erythropoiesis, its immunomodulatory activity is less well defined. We conducted in vitro and in vivo studies on M2 macrophage phagocytotic activity to show that EPO attenuates pro-inflammatory (IL1β, iNOS, and CD68) while augmenting anti-inflammatory (IL10, CD163, and CD206) gene expression. Our data showed that EPO treatment significantly promotes macrophage polarization towards the M2 phenotype with effects on efferocytosis of apoptotic SNSCs in different macrophages populations (BMMØs and PMØs) and following injection of apoptotic SCs into the peritoneum. These experiments provide some evidence that EPO may exert its protective effects in a number of traumatic injuries as a promotor and regulator of post-traumatic inflammation.

In conclusion, we found EPO ameliorated the progression of post-TPNI with effects on nerve function, as well as macrophage phagocytosis. While our work and that of others may shed light on the role EPO plays in improving functional recovery in the traumatized limb [10, 47–51], this work outlines experiments that show effects on macrophage function in parallel with those functional findings. EPO likely improves nerve function after nerve injury via M2 phenotype macrophage phagocytosis of SCs debris with early anti-apoptotic and anti-inflammatory effects.

## Supplementary information

Supplemental Material

Supplemental Figure S1

Supplemental Figure S2

Supplemental Figure S3

Supplemental Figure S4

Supplemental Figure S5

Supplemental Figure S6

Supplemental Figure S7

Supplemental Figure S8

Supplemental Figure S9

Supplemental Figure S10

Supplemental Table S1

aj-checklist
