# Supplementary material for: Erythropoietin promotes M2 macrophage phagocytosis of Schwann cells in peripheral nerve injury
Source: Cell Death Dis. 2022 Mar 16;13(3):245. doi: 10.1038/s41419-022-04671-6 (PMC8927417; doi:10.1038/s41419-022-04671-6)
Supplement: Supplementary file 1 — Supplemental Material [file 41419_2022_4671_MOESM1_ESM.docx]

**Supplemental Information**

**Erythropoietin promotes M2 macrophage phagocytosis of Schwann cells in peripheral nerve injury**

Prem Kumar Govindappa^1^, and John C. Elfar^1,^*

^1^Department of Orthopaedics and Rehabilitation, Center for Orthopaedic Research and Translational Science (CORTS), The Pennsylvania State University College of Medicine, Hershey, PA, 17033, USA

***Correspondence address:**

John C. Elfar, MD

Department of Orthopaedics and Rehabilitation,

Center for Orthopaedic Research and Translational Science (CORTS),

The Pennsylvania State University College of Medicine,

Hershey, PA, 17033, USA

Telephone: +1 717-531-4686

E-mail: [openelfar@gmail.com](mailto:openelfar@gmail.com), [jelfar@pennstatehealth.psu.edu](mailto:jelfar@pennstatehealth.psu.edu)

**Supplemental Legends**

**Supplemental Table S1.** Primer Sequences for qRT-PCR.

**Supplemental Figure S1.** Characterization of BMMØs. (A). Flow cytometry gating strategy of CD11b-APC, F4/80-PE, and dual staining (CD11b-APC + F4/80-PE) for passage 1, BMMØs. (B). The results of BMMØs purity (CD11b + F4/80^+^) from 3 independent experiments are represented in the bar graph. Data are expressed as means ± SEM, n = 3.

**Supplemental Figure S2.** EPO effects on BMMØs cell viability by MTT assay. EPO was not toxic to incubated BMMØs at various concentrations (0.1, 0.25, 1, 5, 10, 25, and 50 IU/mL) at both 24h (A) and 72h (B). Untreated cells were used as controls to calculate the %cell viability. EPO treatment at 24h significantly increases %cell viability as compared to 0.1IU/mL and that was non-significant to 50IU/mL and at 72h. Ordinary one-way ANOVA, Tukey’s multiple comparisons test. Data are expressed as means ± SEM, **P < 0.0021, ***P < 0.0002, and ****P < 0.0001 vs. 0.1IU/mL EPO, n = 4.

**Supplemental Figure S3.** Selection of optimal concentration of EPO against LPS apoptosis induction. (A). Flow cytometry gating strategy of total cells, single cells, and Annexin V-PE (early apoptosis), and 7AAD-PE-Cy5 (late apoptosis) for analysis of the percentage of apoptosis in BMMØs. (B). The result of the percentage of apoptosis following LPS (50ng/mL-upper panel; 100ng/mL-lower panel), and LPS+EPO (5, 25, and 50 IU/mL) treatment at 24h.

**Supplemental Figure S4.** RNA quality of BMMØs. RNA Integrity Number (RIN) values obtained from Agilent 2100 Bioanalyzer System were utilized to test the quality of the RNA samples. (A). The representative electropherogram shows 18S (left) and 28S (right) peaks indicative of RNA integrity of the control, LPS and LPS+EPO treated BMMØs. (B). Electrophoresis of representative samples allows visual inspection of RNA quality of control, LPS and LPS+EPO treated BMMØs.

**Supplemental Figure S5.** Characterization of mouse SNSCs. The identity and purity of SCs were confirmed using IF staining of S100, p75NTR, and MPZ under a fluorescent microscope (ZEISS Apotome 2). The purity of the cultured SCs was analyzed by double positive staining of DAPI with S100/p75NTR/MPZ markers from 3 independent experiments. Each image represents 6 images from 3 independent experiments. Scale bar: 50 μm, n =3.

**Supplemental Figure S6.** Induction of SNSCs apoptosis using H_2_O_2_. (A). IF staining of Annexin V/DAPI of SNSCs following induction by 250μM of H_2_O_2_ and the results are represented in the bar graph (B). Each image represents 9 images from 3 independent experiments (C). Flow cytometry gating strategy of total cells, single cells, and Annexin V-PE (early apoptosis), and 7AAD-PE-Cy5 (late apoptosis) for analysis of the percentage of apoptotic SNSCs and the results are represented in the bar graph (D). Data are expressed as means ± SEM, n = 3.

**Supplemental Figure S7.** Induction of SNSCs apoptosis using LPS. (A). Flow cytometry gating strategy of total cells, single cells, and (B) Annexin V-PE (early apoptosis), and 7AAD-PE-Cy5 (late apoptosis) for analysis of %apoptosis in SNSCs.

**Supplemental Figure S8.** Characterization of mouse PMØs. (A). Flow cytometry gating strategy of CD11b-APC, F4/80-BV421, and dual staining (CD11b-APC+F4/80-BV421) for PMØs. (B). The results of PMØs purity (CD11b + F4/80^+^) are represented in the bar graph. Data are expressed as means ± SEM, n = 3.

**Supplemental Figure S9.** Gating strategy of BMMØs-efferocytosis of apoptotic SNSCs. (A). Flow cytometry gating strategy of total cells, single cells, and dual staining of CD11b-APC. and F4/80-BV421. (B). Gating strategy of efferocytosis of apoptotic SNSCs (PKH26-PE positivity in CD11b+F4/80^+^cells).

**Supplemental Figure S10.** EPO effect on M2 phenotype macrophages during phagocytosis of apoptotic SNSCs. EPO treatment (5000 IU/kg/IP, immediately after surgery and on post-surgery day1and 2) moderately increases CD206^+^ (dual staining of CD11b and CD206) peritoneal macrophages as compared to saline treatment (normal saline, 0.1ml/IP/mouse) following intraperitoneal injection of apoptotic SNSCs in mice. Unpaired t-tests. Data are expressed as means ± SEM, n = 3/group.
