# Supplementary figures and images for: Erythropoietin promotes M2 macrophage phagocytosis of Schwann cells in peripheral nerve injury
Source: Cell Death Dis. 2022 Mar 16;13(3):245. doi: 10.1038/s41419-022-04671-6 (PMC8927417; doi:10.1038/s41419-022-04671-6)

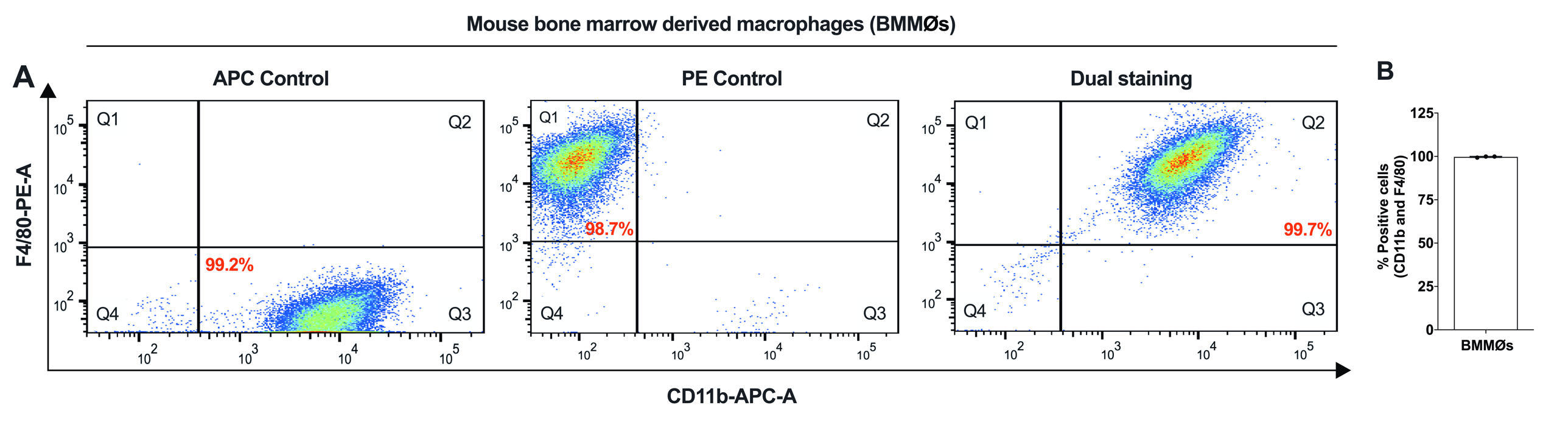

Supplement: Supplementary file 2 — Supplemental Figure S1 [file 41419_2022_4671_MOESM2_ESM.tif]

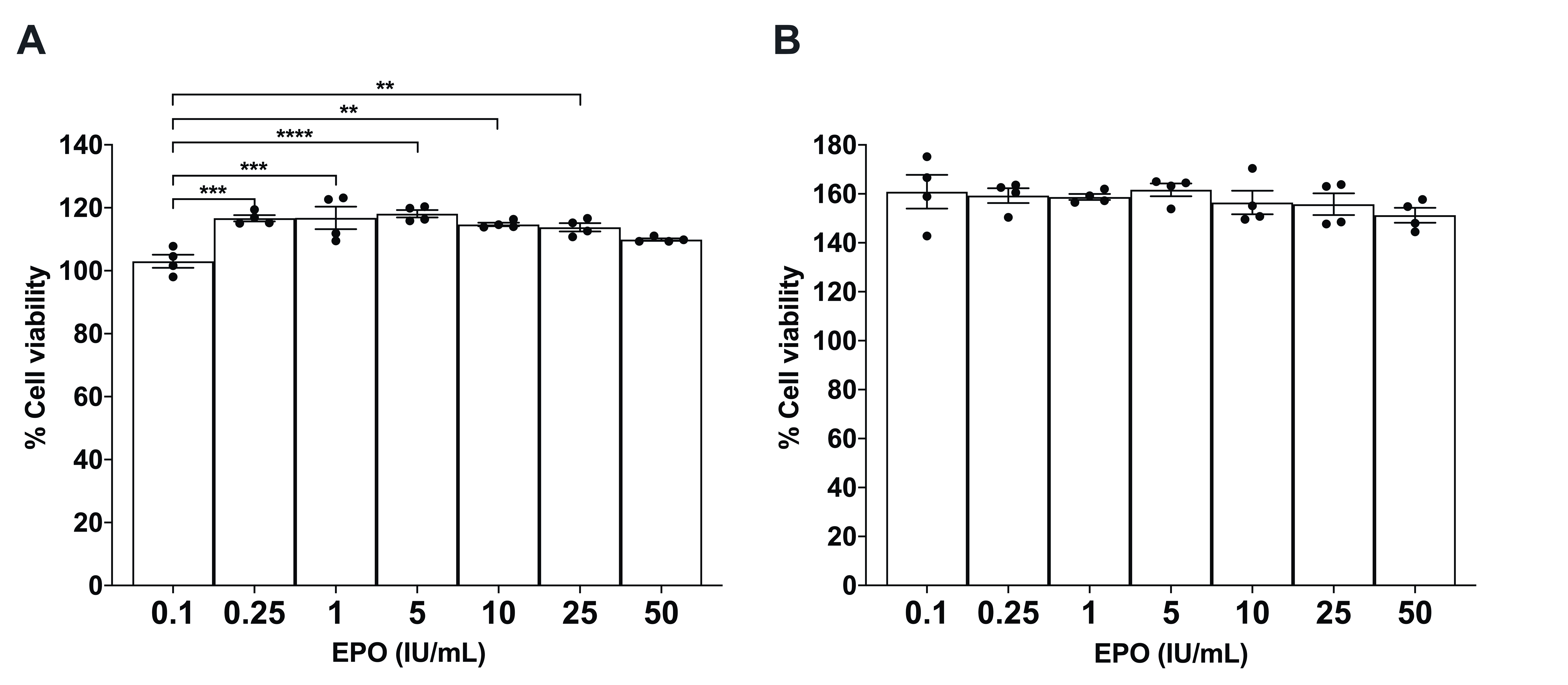

Supplement: Supplementary file 3 — Supplemental Figure S2 [file 41419_2022_4671_MOESM3_ESM.tif]

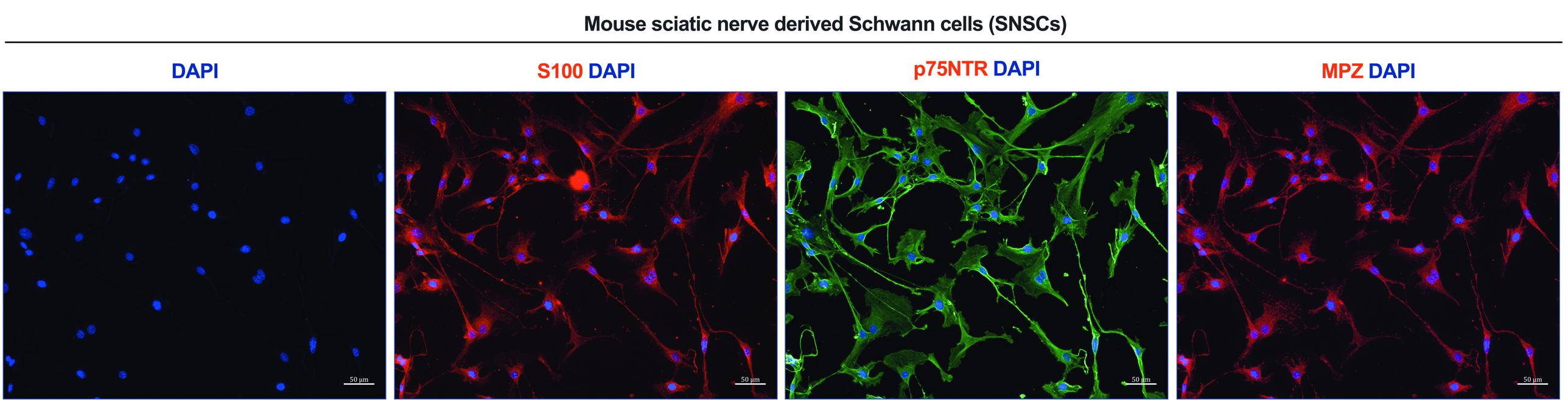

Supplement: Supplementary file 6 — Supplemental Figure S5 [file 41419_2022_4671_MOESM6_ESM.tif]

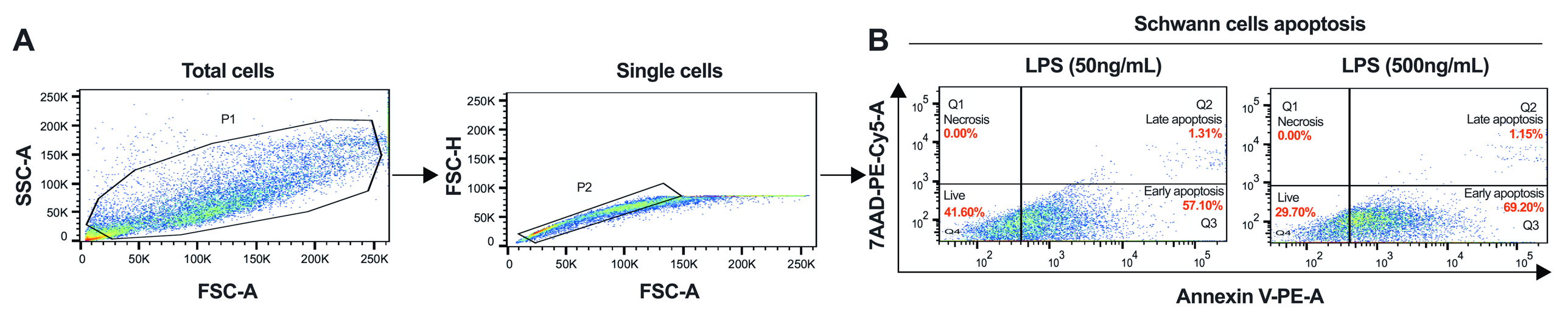

Supplement: Supplementary file 8 — Supplemental Figure S7 [file 41419_2022_4671_MOESM8_ESM.tif]

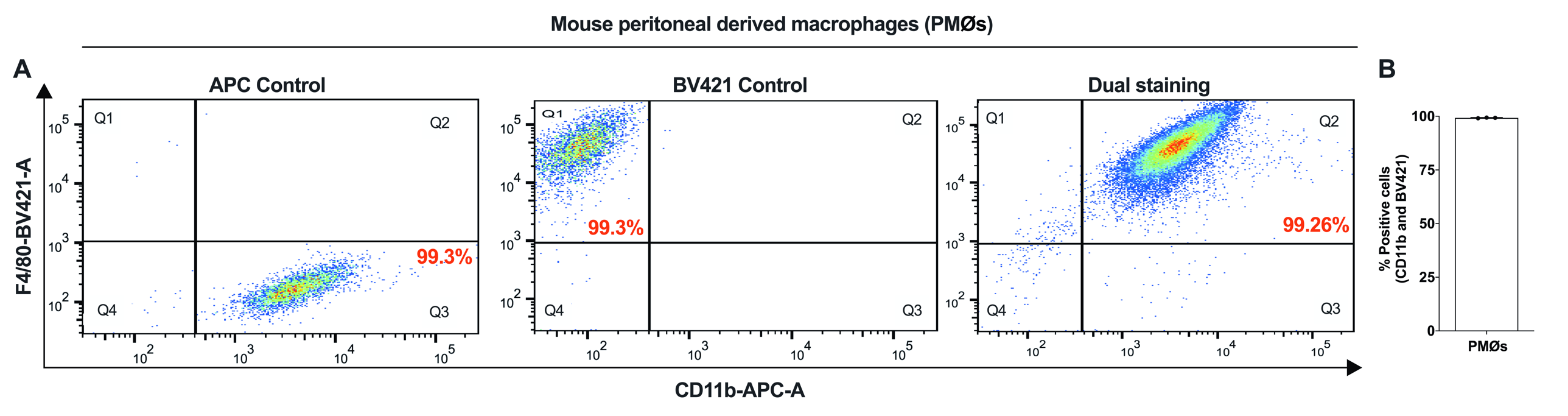

Supplement: Supplementary file 9 — Supplemental Figure S8 [file 41419_2022_4671_MOESM9_ESM.tif]

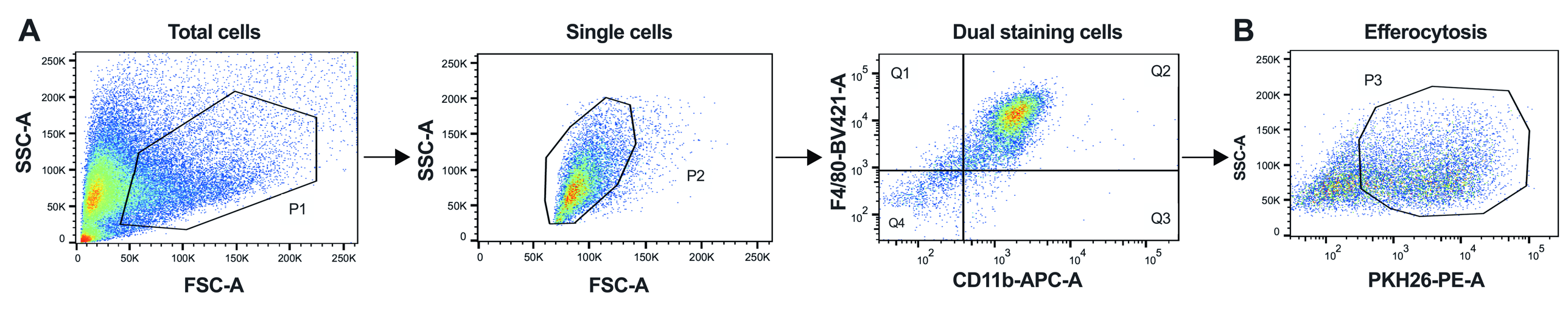

Supplement: Supplementary file 10 — Supplemental Figure S9 [file 41419_2022_4671_MOESM10_ESM.tif]

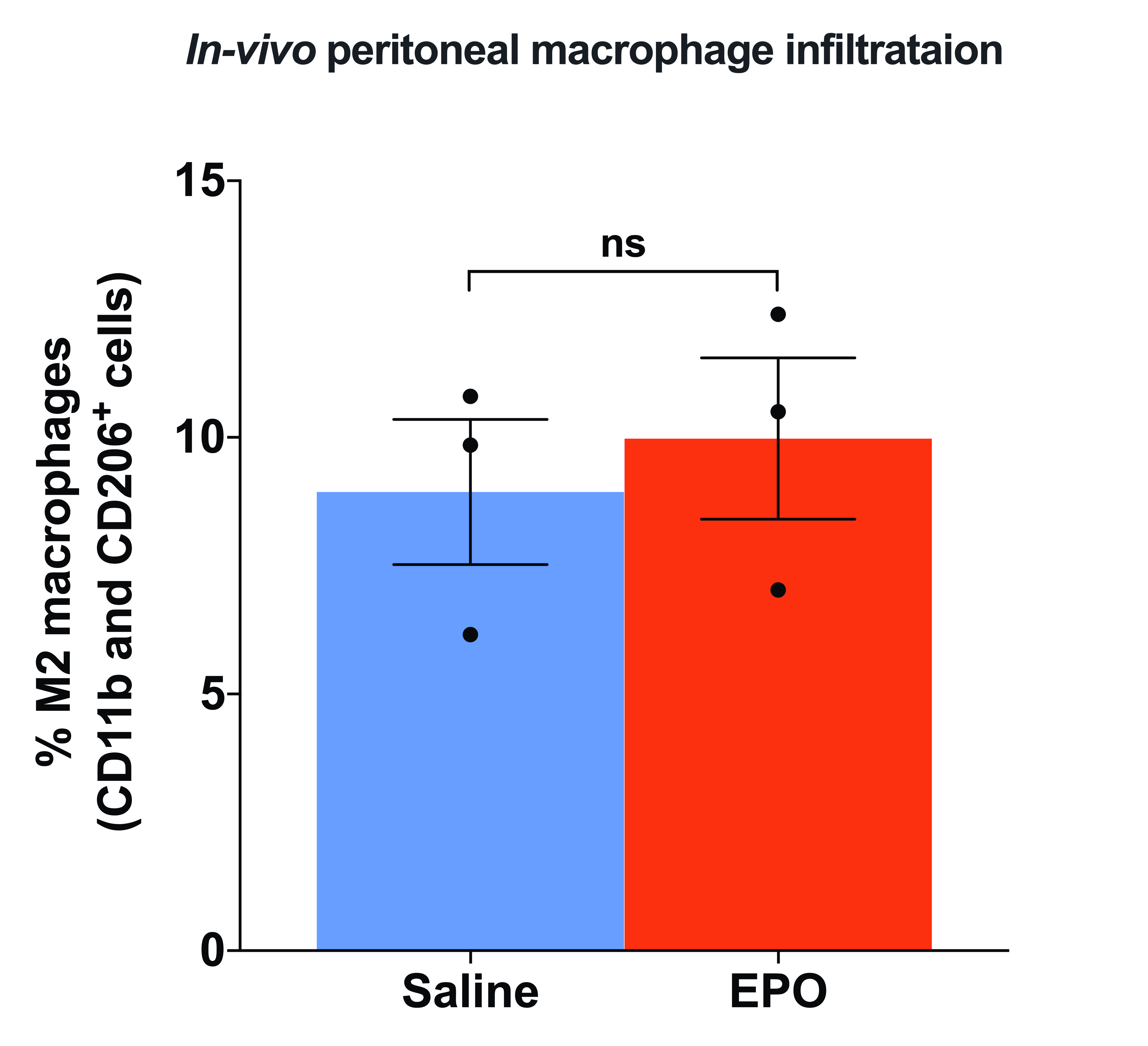

Supplement: Supplementary file 11 — Supplemental Figure S10 [file 41419_2022_4671_MOESM11_ESM.tif]
